# Designing of cardanol based polyol and its curing kinetics with melamine formaldehyde resin

**DOI:** 10.1080/15685551.2016.1231030

**Published:** 2016-10-21

**Authors:** Dinesh Bapurao Balgude, Anagha Shyamsunder Sabnis, Swapan Kumar Ghosh

**Affiliations:** ^a^ Department of Polymer and Surface Engineering, Institute of Chemical Technology, Mumbai, India; ^b^ Research and Development Centre, Nova Surface-Care Centre Pvt. Ltd., Mumbai, India

**Keywords:** Natural resource, cardanol polyol, curing kinetics, industrial coatings, performance properties

## Abstract

Commercially used industrial baking enamels consist of alkyd or polyester resin with melamine formaldehyde. These resins are mainly derived from fossil resources. Considering growing environmental legislation regarding use of petroleum based raw materials, utilization of renewable resources to synthesize various chemistries can be the only obvious option as far as academia and industries are concerns. The present work deals with exploration of one of the natural resources (Cardanol) for polyol synthesis, its characterization (FTIR and NMR) and its curing behavior with melamine formaldehyde resin by differential scanning calorimetry (DSC). The optimized formulations from DSC study were further evaluated for general coating properties to study the suitability of developed polyol for industrial coating application. The experimental studies revealed that melamine content in the curing mixtures and thereby developed crosslinking density played an important role in deciding the coatings properties.

## 1. Introduction

The composition for the industrial baking enamels mainly consists of base resin (alkyd/acrylic/polyester) and crosslinker (melamine formaldehyde resin) along with p-toulene sulphonic acid as a catalyst to accelerate the curing mechanism. Being aliphatic in nature, the base resin (alkyd/polyester) gives excellent flexibility while the heterocyclic ring of melamine resin contribute to hardness and chemical resistance properties.[1] The base resins are mainly derived from petroleum mass. Though these resins have played an important role in the conventional market, their uses have been overshadowed due to stringent environmental legislation, exponential rising prices and uncertainty in the availabilities of petroleum based feed stocks. Considering these issues, the exploration of renewable resources for polymer/resin syntheses is the only obvious option.[2] Till date numbers of researchers have reported all sorts of possible applications of renewable resources such as resin synthesis, adhesives, paints, coatings, composites etc. through various chemical modifications.[3–9] As far as industrial coatings are concerned, polyesters are the most widely used resins in coating industry. They are usually produced by polycondensation of dibasic acid and di-hydroxy compounds and are mainly characterized by presence of ester linkage (COO–). The most common renewable monomers used for polyesters are sugar alcohols, lignin, tannin, shellac, rosin etc. These materials have proved to be excellent coating materials as per the earlier reports.[10–14] However, there exists compound like Cashew Nut Shell Liquid (CNSL), which can be used as a possible substitute for petroleum based feed stocks due to its availability, sustainability, cost effectiveness, and reactive functionalities.

CNSL, an agricultural waste of cashew nut tree contains number of useful phenolic derivatives with meta-substituted saturated/unsaturated hydrocarbon long chain. These reactive functionalities make them suitable for number of polymerization reactions through addition as well as condensation mechanisms. Cardanol, one of the key components of CNSL constituents have also been reported as a potential raw material for synthesis of various chemistries such as epoxy, alkyds, phenolics, polyols etc.[15] Cardolite, one of the leaders in cashew technology has modified the cardanol molecule in order to incorporate additional functionalities such as epoxy, amine, vinyl etc. Some of the modified products include Diglycidyl ether of cardanol (Cardolite NC-514), Mono-glycidyl ether of cardanol (Cardolite NC-513), Amine functional curing agent based on cardanol with a generic name of phenalkamines (NC-540, NC-557, NC-566 and so on) and phenalkamide (LITE 3060). By late 70’s, these modified cardanol found extensive scope in various applications when used as such and/or through further chemical modifications and till date it is one of the most widely used natural resources for various industrial applications.

The aim of the present work was to design and characterize cardanol based polyol. Further, the synthesized polyol was studied for its curing behavior with melamine formaldehyde resin by differential scanning calorimetry (DSC). The curing mixtures at optimized curing temperature and curing time obtained from DSC study was evaluated for general coating properties such as mechanical, chemical and solvent resistance, anti-corrosive and thermal properties.

## 2. Experimental

### 2.1. Materials

The main raw materials used was cardanol based diglycidyl ether, Cardolite NC-514 (Epoxy equivalent weight = 490–535 g/eq), provided by Cardolite Speciality Chemicals Ltd., Manglore, India. The reagents used like tartaric acid, anhydrous sodium sulphate, acetic anhydride, pyridine, triphenyl phosphine (TPP), dimethyl formamide (DMF), tertabutyl ammonium bromide (TBAB), sodium hydroxide, phenolphthalein indicator, oxalic acid, p-toluene sulfonic acid (p-TSA), potassium hydroxide etc. were lab grade chemicals procured from SD Fine Chemicals Ltd., Mumbai and were used as supplied. Hexamethoxy methylmelamine (HMMM) was obtained from Shalimar Paints Ltd, Nasik, India.

### 2.2. Synthesis of cardanol based polyol

Commercially available cardanol based diglycidyl ether (NC-514) was reacted with multifunctional acid (tartaric acid) to incorporate the additional functionalities in the polymer backbone. Synthesis of cardanol based polyol involved ring opening of NC-514 with functional acid in presence of ring opening catalyst (triphenylphosphine). At first, epoxy cardanol (NC-514) and tartaric acid along with phase transfer catalyst (TBAB = 0.5 wt%) and solvent (DMF = 20 wt%) was charged in a molar ratio of 1:2.1 (NC-514: tartaric acid) in a 3-neck round bottom flask fitted with thermometer pocket, water condenser, and mixed using a motor driven stirrer at temperature (95–100 °C) for ½ hr. The ring opening catalyst (Triphenylphosphine = 1 wt%) was then added and the reaction was continued at same temperature. The reaction was monitored till desired acid value.

**Figure 1.**
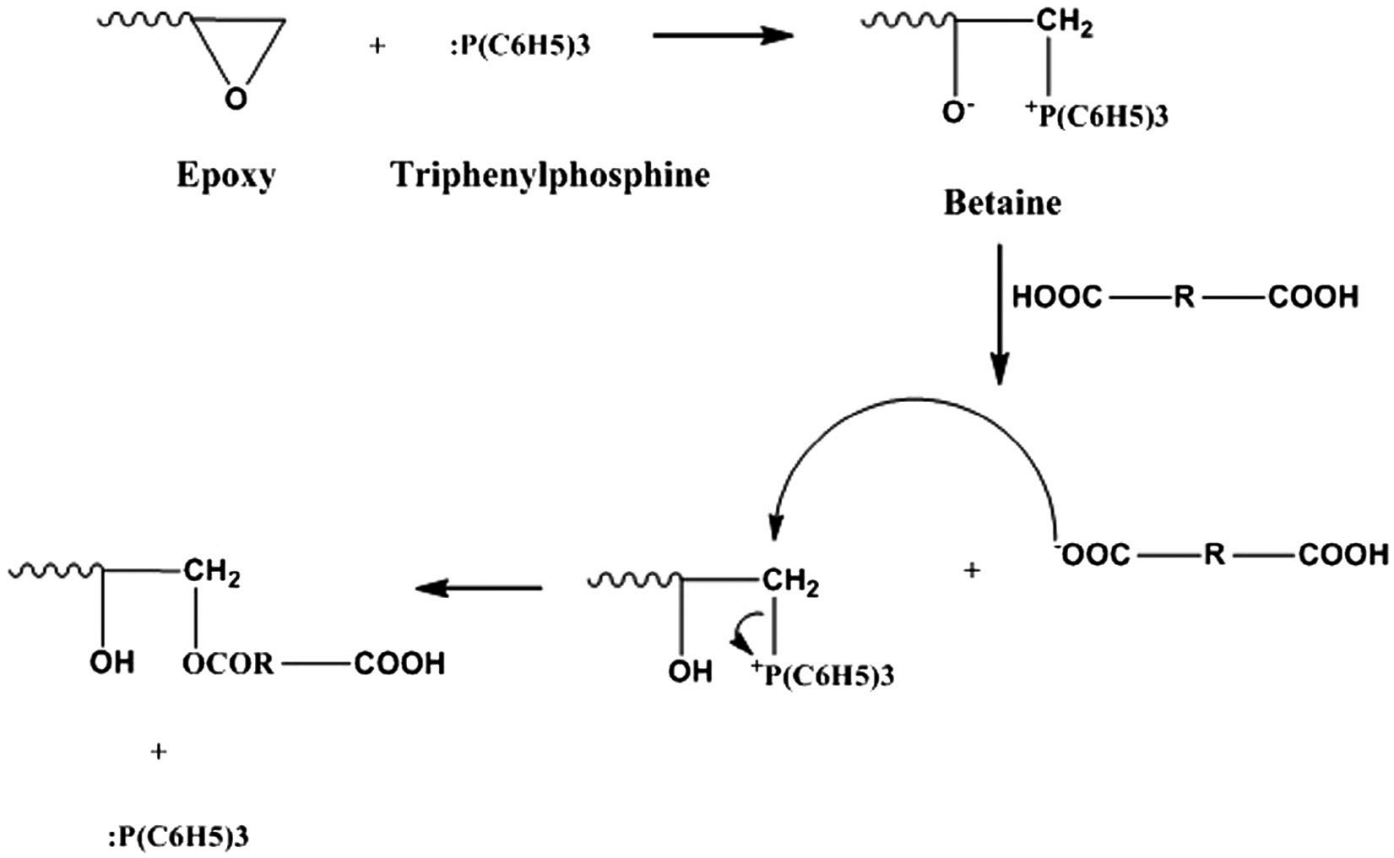
Oxirane ring opening reaction mechanism by carboxylic acid.

**Figure 2.**
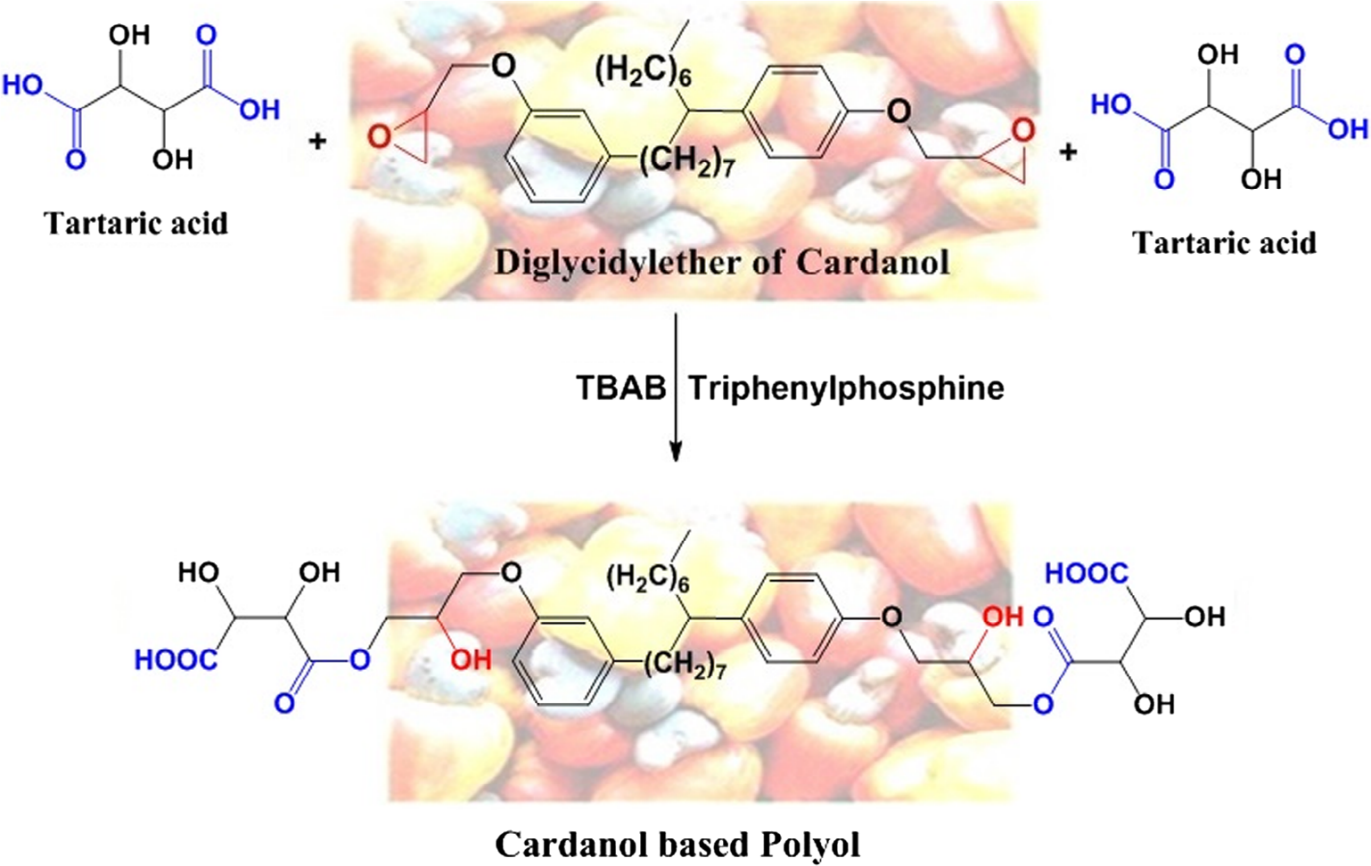
Schematic representation of synthesis of cardanol based polyol.

The ring opening reaction mechanism of epoxy with TPP involves neucleophilic attack by triphenylphosphine on epoxy ring, producing a betaine. Proton abstraction from acids yields the carboxylic anion, forming a phosphonium salt. The carboxylic anion reacts with the electrophilic carbon attached to the positive phosphorus, regenerating the catalyst.[16] The reaction mechanism is shown in Figure 1. After reaching the desired acid value, the reaction was cooled down to room temperature (28 °C) and the product obtained was washed with lukewarm water to remove unreacted acid till neutral pH. The water was distilled off under reduced pressure followed by passing the product over anhydrous sodium sulphate to remove the water traces. The purified product was further characterized for its physical and chemical properties. Figure 2 shows schematic representation of synthesis of cardanol polyol.

### 2.3. Methods

#### 2.3.1. Physical and chemical characterizations of developed polyol

The synthesized polyol was characterized for its physical properties (i.e. color, % non-volatile matter, viscosity, specific gravity) [17] and chemical properties (such as epoxy value,[18] acid value [19] and hydroxyl value [20]) as per the ASTM standards.

#### 2.3.2. Spectroscopic measurements

Further, the polyol was characterized for presence of functional groups by Fourier Transform Infrared Spectroscopy (FTIR) on MIRacle 10, SHIMADZU Instrument as per ASTM E-1252. Forty five scans were recorded for each sample in the spectra range from 4000 to 500 cm^−1^. The chemical structure of the polyol developed was further confirmed by NMR analysis on Bruker Biospin (Avance AV500WB, Germany) spectrometer at 400 MHz using deuterated chloroform as solvent and tetramethylsilane (TMS) as an internal standard.

#### 2.3.3. Curing kinetic study

##### 2.3.3.1. Preparation of resin mixtures

The developed polyol was mixed with HMMM at various concentrations of HMMM (from 10 to 30% based on dried mass) in order to optimize the curing behavior. p-TSA (1 wt%) was used as a catalyst to accelerate the reaction between polyols and HMMM. Polyol and requisite amount of HMMM along with p-TSA were homogenized at room temperature. The prepared resin mixtures were investigated for their curing behavior by differential scanning calorimetry (DSC). The designations of the prepared resin mixtures are reported in Table 1.

**Table 1.** Designations and curing formulations of prepared resin mixtures.

Designation	Cardanol polyol (wt%)	HMMM resin (wt%)	pTSA catalyst (wt%)
70–30	70	30	1
75–25	75	25	1
80–20	80	20	1
85–15	85	15	1
90–10	90	10	1

##### 2.3.3.2. Curing behavior of cardanol polyol with MF resin by DSC

The curing mechanism of polyol and melamine formaldehyde resin involved two main reactions; hetero-polycondensation (condensation between functional groups of two different resins i.e. polyol and HMMM) and homo-polycondensation (condensation between functional groups of same resins i.e. only HMMM) which take place simultaneously. Curing conditions should be selected to favor hetero-polycondensation resulting in the formation of three-dimensional network. The type of polycondensation reaction mainly depends on type of amino-aldehyde resins which differ from one another in type of functional groups, degree of polymerization and the type of alcohol used for etherification. In our study, we have used hexa functional methoxy melamine formaldehyde resin (HMMM) which is one of the most frequently used curing agents for hydroxyl functional resins. During hetero-polycondensation with hydroxyl and carboxyl functional cardanol polyol, trans-etherification and trans-esterification takes place respectively.[21] The possible curing reactions are represented by Equations (1) and (2).

(1) $${\text{R}}^{\prime }-\text{N}-{\text{CH}}_{2}{\text{OCH}}_{3}+\text{R}-\text{OH}\to {\text{R}}^{\prime }-\text{N}-{\text{CH}}_{2}\text{OR}+{\text{CH}}_{3}\text{OH}$$

(2) $${\text{R}}^{\prime }-\text{N}-{\text{CH}}_{2}{\text{OCH}}_{3}+\text{R}-\text{COOH}\to {\text{R}}^{\prime }-\text{N}-{\text{CH}}_{2}\text{OCOR}+{\text{CH}}_{3}\text{OH}$$

Curing behavior of cardanol based polyol with melamine formaldehyde resin was studied by differential scanning calorimetry (DSC) using DSC-60 SCHIMADZU INSTRUMENTS. The sample was placed (around 5 mg) in a DSC hermetically sealed pan and freed of solvent in a vacuum oven at ambient temperature before the sample was encapsulated. The sample was mounted in the instrument and scanned. The Ozawa iso-conversional method [22,23] was applied at three heating rates (5, 10 and 15 °C/min) in a scanning temperature range from 5 to 260 °C in dynamic region to calculate the kinetic parameters of curing mixtures.

The Ozawa method is based on following expression:

(3) $$\log\beta ={\text{A}}^{\prime }-0.4567\frac{E_{\text{a}}}{RT}$$

where, *β* is the heating rate (°C/min), *E*_a_ is the activation energy (J/mol), *R* is the gas constant (8.314 J mol^–1^ K^–1^), *T* is the temperature (K) and *A*′ can be expressed as:

(4) $$A^{\prime }=\log\left[\frac{k_{\text{o}}E_{\text{a}}}{Rg\left(\alpha \right)}\right]-2.315$$

where, *α* is the apparent degree of curing reaction, *k*_o_ is the pre-exponential factor of Arrhenius law and *g*(*α*) is a function of curing degree:

(5) $$g\left(\alpha \right)=\int_{0}^{\alpha }\frac{d\alpha }{f\left(\alpha \right)}=\int_{0}^{t}k_{\text{o}}e^{-\frac{E_{\text{a}}}{RT}}dt\;=k_{\text{o}}e^{-\frac{E_{\text{a}}}{RT}}\;t$$

From *A*′ and *E*_a_ values obtained from Equation (3) for different apparent curing degrees are possible to determine a new constant, *A*, which can be written as:

(6) $$A=\ln\left[\frac{g(\alpha )}{\left(k_{\text{o}}\right)}\right]$$

Further, the curing mixtures were scanned at different isothermal temperatures (120, 130, 140 and 150 °C) for 30 min to study the degree of curing reaction at particular temperature. The curing temperatures in present work were selected according to the usual range employed in making baking enamels. After each isothermal curing, the uncured resin mixture content was determined by heating the sample (from the temperature of isothermal curing to 250 °C) in the dynamic thermal range at the heating rate of 10 °C/min.[24] The degree of curing reaction (*α*) was obtained from DSC measurements as proportional to the enthalpies of the exothermic events at a particular isothermal temperature. The degree of curing reaction (*α*) is expressed as;

(7) $$\alpha =\;\frac{\int_{T0}^{Tt}\left(\frac{dH_{t}}{dT}\right)dT}{\int_{T0}^{T}\left(\frac{dH}{dT}\right)dT}$$

where, *dH*_*t*_/*dT* is the reaction enthalpy at time (*t*), *dH/dT* is the total reaction enthalpy, *T*_0_ is the initial curing temperature, *T*_*t*_ is the curing temperature at time (*t*), *T* is the final curing temperature.

From the results of apparent degree of curing reaction vs. temperature, the isothermal temperature at which maximum degree of curing obtained was finalized and then the values of degree of curing at each time were calculated to obtain the conversion degree vs. curing time at different ratios of curing mixtures at optimized temperature using Equation (8).

(8) $$\ln\left(t\right)=A+\frac{E_{\text{a}}}{RT}$$

The parameter *A* of the Equation (8) is the same of the Equation (6), accepting equal kinetics in dynamical and isothermal experiments. In both equations, *A* is a function of *g*(*α*) and *k*_o_ and consequently, only a function of apparent curing degree.

The kinetic parameter such as reaction order and the rate constant of curing mixtures were calculated by isothermal DSC data at optimized temperature. The rate of chemical reaction, *dα/dt*, can be expressed as a function of the degree of conversion using general formulation:

(9) $$\frac{d\alpha }{dt}=k\;\left(T\right)f\left(\alpha \right)$$

where *t* is the time, *T* the temperature, *k* is the rate constant and *f*(α) is a function of degree of conversion. Assuming that the curing reaction follows *n*th order kinetics *f*(α) has the form [25]: *f*(α) = (1 – *α*)^*n*^. Substituting *f*(α) in Equation (9) and applying logarithmic, the equation can be written as;

(10) $$\log\left(\frac{d\alpha }{dt}\right)=n\log\left(1-\alpha \right)+\log k$$

The value of reaction order (*n*) and rate constant (*k*) was calculated by plotting log (*dα*/*dt*) vs. log (1 – *α*).

#### 2.3.4. Coating film characterizations

The curing mixtures at optimized curing temperature and time obtained from DSC study were evaluated for general coating properties. The curing mixtures were diluted with mixture of solvents (xylene: butanol = 80:20 v/v) to adjust the application viscosity at 40% non-volatile matter. The diluted curing mixtures were applied onto prepared mild steel substrate by conventional air assisted spray gun according to ASTM D 4708-99. The coated substrates were allowed to air dry for 10–15 min and were then placed in an air circulating oven to cure at optimized temperature and time which was obtained from DSC study. The completely cured resin mixtures were then evaluated for general coating properties as follows.

##### 2.3.4.1. Mechanical and optical properties

The completely cured coatings were evaluated for their optical properties (gloss) by Digital Glossometer (Micro-Tri-Gloss, Byk Instrument). Applied coatings were evaluated for adhesion properties by cross cut adhesion with commercial cellophane tape (25-mm wide semitransparent tape manufactured by Permacel, New Brunswick, NJ 08903) according to ASTM D-3359. Pencil and scratch hardness of the coating were measured on hardness tester according to ASTM D-3363 and ISO 1518-2 respectively. Flexibility of the coatings was tested by conical mandrel method as per ASTM D-522. The load distribution properties of the coating were tested by impact tester as per ASTM D-2794. Impact resistance was measured on the impact tester with maximum height of 100 cm and load of 1.86 kg.

##### 2.3.4.2. Chemical and solvent resistance properties

The film integrity of the coatings was evaluated by double rub method as per ASTM D-4752. MEK and xylene were used as solvents to evaluate the film integrity. Similarly, the chemical resistance properties of the coatings were evaluated by dipping the coated panels in 5% HCl and 5% NaOH for 24 h respectively. Completely cured coatings were tested for their hydrolytic stability as per ASTM-B-1308. The coated panel was immersed in boiling water for 24 h. After chemical resistance, solvent resistance and hydrolytic stability tests were performed, the coated panels were again evaluated for degree of adhesion and inspected visually for any blisters and cracks.

##### 2.3.4.3. Anti-corrosive properties

The corrosion resistance properties of the cured coatings were evaluated using Tafel polarization studies and electrochemical impedance spectroscopy (EIS) on ACM Instrument (Gill AC Serial No. 1641). Three-electrode system was used, in which saturated calomel electrode, platinum electrode and coated panels were reference, counter and working electrode respectively. Electrode surface area exposed to testing solution (5 wt% NaCl) was 1 cm^2^ in all the cases. All the electrochemical measurements were carried out at room temperature (27 ± 1 °C) in 5 wt% NaCl solution.

##### 2.3.4.4. Determination of insoluble sol fraction

The insoluble sol fraction was determined by extraction method using mixture of solvents (xylene: dimethyl formamide: acetone = 50: 25: 25 by v/v). The prepared resin mixtures were applied on glass panel at thickness of 50–60 μm and were allowed to standby at room temperature in order to flash off the solvents. After that the panels were cured at various isothermal temperatures (120, 130, 140 and 150 °C) for 30 min. Then weight of the panels with cured film was measured (*w*_0_) and panels were put in solvent mixture for 24 h at ambient temperature in order to extract the insoluble sol fraction. After that the glass panels with insoluble sol fraction was vacuum dried and their weights were noted (*w*_t_) The content of sol fraction was calculated from the difference in mass of sample before (*w*_0_) and after extraction (*w*_t_).[26]

##### 2.3.4.5. Thermal properties

The glass transition temperature and thermal degradation temperature was evaluated by Differential Scanning Calorimetry (DSC-60, SHIMADZU Instrument) and Thermogravimetric Analysis (TGA-51, SHIMADZU Instrument) at a heating rate of 10 °C/min under nitrogen atmosphere. DSC was performed by heating the sample from −50 to 150 °C at a scan rate of 10 °C/min and a modulated frequency of −0.5 °C every 40 s. The test was conducted under a N_2_ flow of 10 ml/min. For TGA analysis, 5–6 mg of samples were kept in an alumina pan and scanned at a heating rate of 10 °C/min up to 600 °C in a nitrogen atmosphere with 10 ml/min nitrogen gas flow rate.

## 3. Result and discussion

### 3.1. Physical and chemical characterizations of developed polyol

The color of synthesized polyol was checked on Gardener Color Scale (BYK Instrument) and was observed to be <12. The specific gravity and Brookfield viscosity was recorded at 80% NVM at room temperature (27.9 °C) by weight per litre cup and Brookfield viscometer (DV-II+Pro, Model No. LVDV-II+P) respectively. The prepared polyol was evaluated for unreacted epoxy content, acid value and hydroxyl value by titration method. The completion of the reaction was confirmed by decreased epoxy values of the final purified product. The epoxy value, as measured by volumetric titration method, was observed to be in the range of 0.1–0.3 mmol/kg confirming that the reaction was complete. All the physical and chemical characterization values of cardanol polyol are reported in Table 2.

**Table 2.** Physical and chemical properties of cardanol polyol.

Properties	Observed values
Color [gardener]	<12
% Non-volatile matter	80.56%
Brookfield viscosity [cP], Spindle no. S62	4809 cP
Specific gravity	1.054
Theoretical acid value	88 mg of KOH/gm
Practical acid value	90 mg of KOH/gm
Theoretical hydroxyl value	263 mg of KOH/gm
Practical hydroxyl value	276 mg of KOH/gm

### 3.2. Spectroscopic measurements

**Figure 3.**
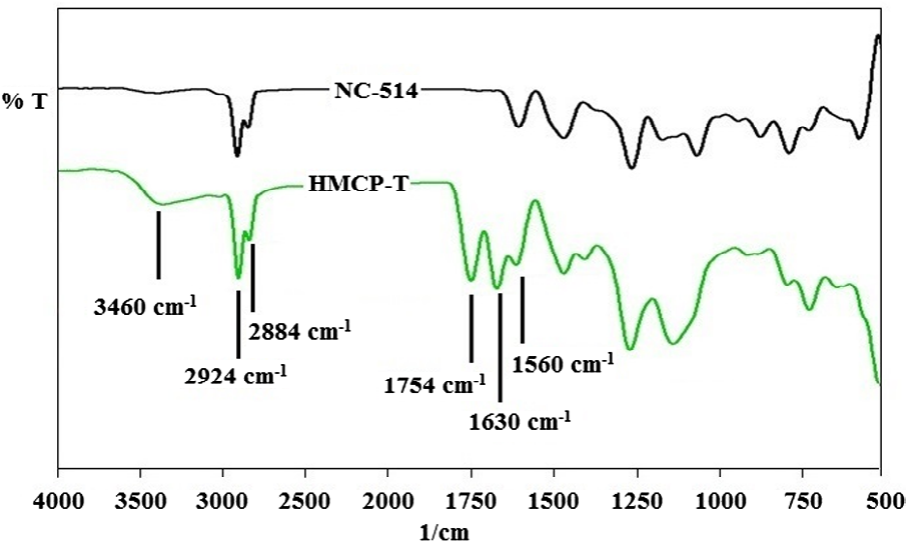
FT-IR spectra of cardanol based polyol.

The synthesized cardanol polyol was further characterized by FTIR spectroscopy to confirm the presence of functional groups as shown in the Figure 3. The peak intensity of absorption band around 1754 cm^−1^ due to C=O stretching confirmed the presence of carboxylic group (–COOH) in the structure, whereas, the peak around 3460 cm^−1^ denoted the existence of hydroxyl (–OH) groups. The transmission band at 2924 and 2884 cm^−1^ were attributed to aliphatic and aromatic C–H stretchings respectively. Aromatic C=C bonds were confirmed by band at 1560–1630 cm^−1^. Further the ring opening reaction of epoxy group was confirmed to be completed by absence of absorption band at 909 cm^−1^ in the spectrum which is a characteristic peak of C–O–C linkage in epoxy ring.[27]

**Figure 4.**
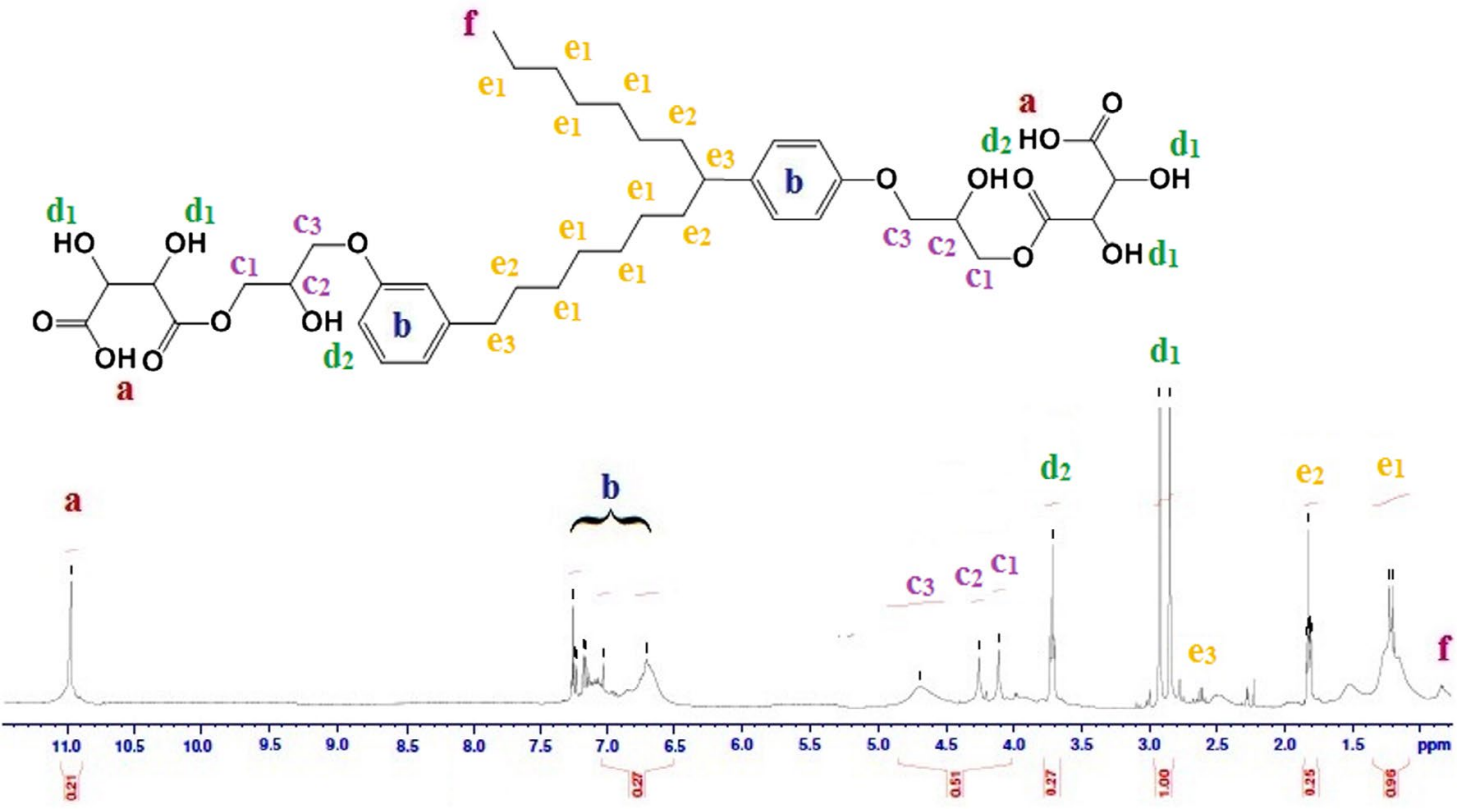
^1^H-NMR of cardanol based polyol.

Further the developed polyol was characterized by NMR spectroscopy to confirm the completion of reaction as shown in Figure 4. It can be seen from the figure that the polyol showed its characteristic peaks. The chemical shift at 0.92 ppm can be attributed to terminal methyl protons from long aliphatic chain of cardanol. The signals obtained at 1.25, 1.7 and 2.7 ppm could be attributed to the methylene protons from aliphatic chains. The alcoholic protons adjacent to carbonyl group could be confirmed by the signals near 2.8–2.9 ppm, whereas signal at 3.75 ppm could be attributed to secondary hydroxyl formed after ring opening reaction. The proton from α-carbon atom of alcoholic groups could be observed with the peaks at 4.35 whereas protons from β-carbon atom of alcoholic groups could be observed at 4.31 and 4.65 ppm. The chemical shifts at 6.68–7.35 can be assigned to proton form benzene rings, whereas shift at 11 ppm can be due to proton from carboxylic groups.[28] The results obtained confirmed successful formation of polyol.

### 3.3. Curing kinetic study

**Figure 5.**
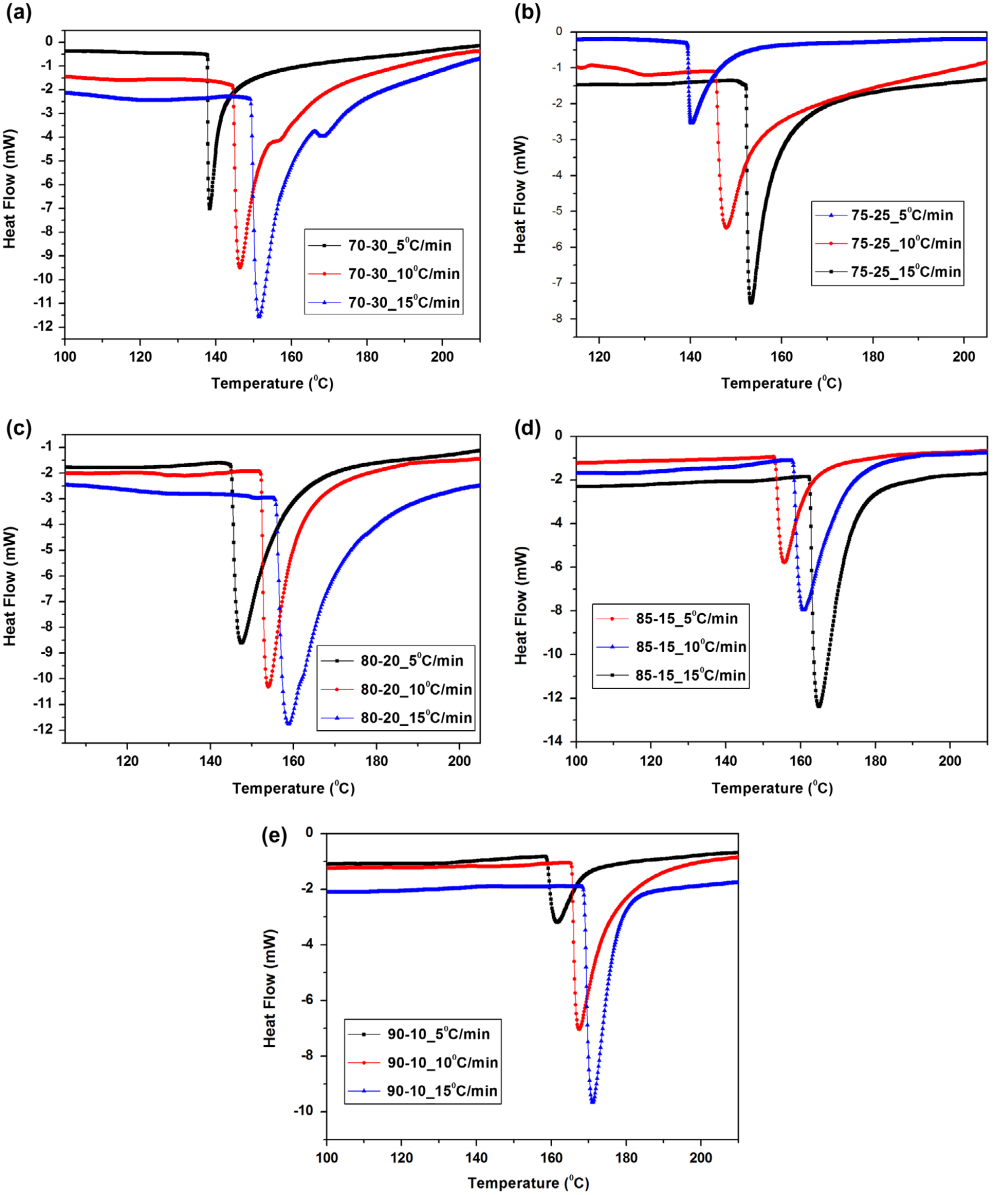
Dynamic DSC curves of curing of cardanol polyol/melamine formaldehyde resin at different heating rate (a) 70–30 (b) 75–25 (c) 80–20 (d) 85–15 (e) 90–10.

**Figure 6.**
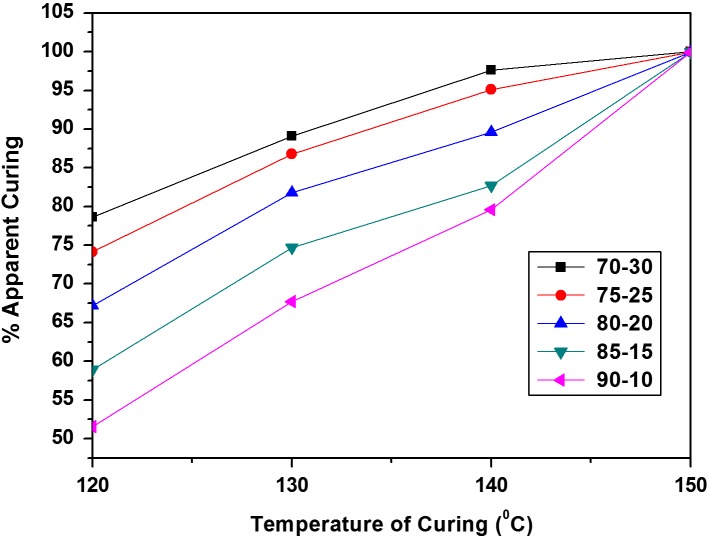
Apparent degree of curing vs. temperature of curing of resin mixtures.

**Figure 7.**
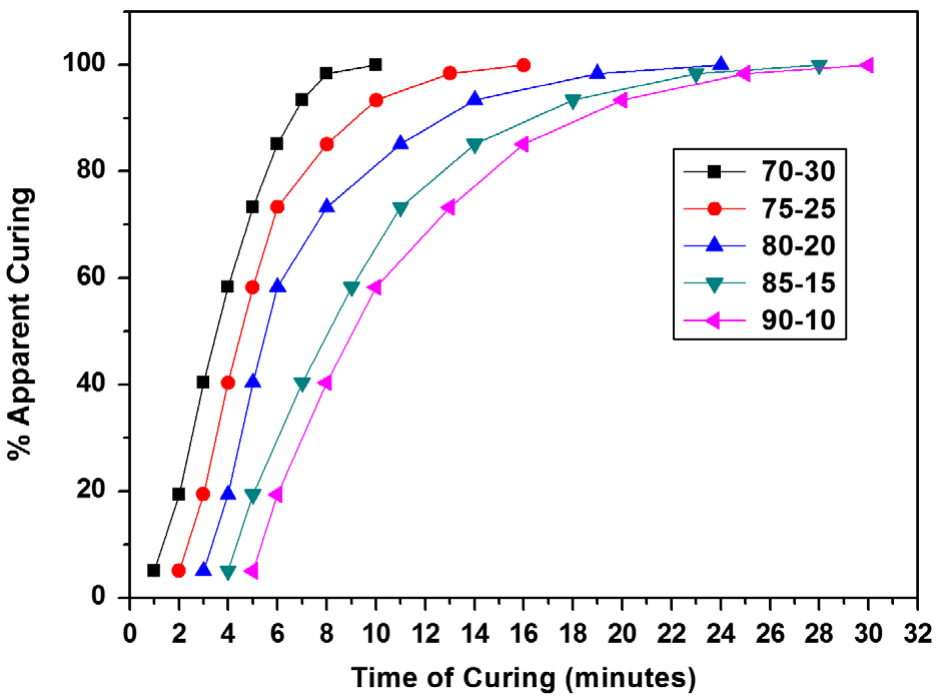
Apparent degree of curing vs. time of curing of resin mixtures at optimized temperature.

The dynamic DSC scans of various resin mixtures at different heating rates are given in Figure 5(a)–(e), it is seen that a single peak is obtained in all the thermograms. The temperature of the maximum in the curing curves increases with decreasing quantity of melamine resin in the resin mixtures and with increasing heating rate. The values of constant *A*′ and activation energy *E*_a_ at different apparent curing degrees of all curing mixtures were calculated through Ozawa method (Equation 3). Activation energy values at each apparent degree of cure were calculated from the slope of the plot of (log *β*) vs. (1/*T*). At the same time values of *A*′ have been obtained from the intercept in each case (Equation 3). This data was then fitted to Equation (4) to obtain parameter *A*. The activation energies (*E*_a_) and constants (*A* and *A*′) for the cardanol polyol/melamine formaldehyde resin mixtures curing at different degrees of conversion are shown in Tables 3 and 4 respectively. Activation energy showed significant dependence on the apparent degree of curing, in a direct proportion, which was in agreement with literature data for other thermosetting resins.[29,30] This is a characteristic of a curing process of thermosetting resins [29–31] irrespective of whichever model is used (Ozawa or Kissinger). Further, from isothermal curing, it was observed that degree of curing depends on temperature of curing, increasing with temperature as shown in Figure 6. At isothermal temperature of 150 °C, the maximum conversion was obtained. This isothermal temperature was selected to calculate the degree of curing at each time of interest from DSC scan of isothermal data which is presented in Figure 7, which shows the conversion degree vs. curing time for different ratios of resin mixtures.[29]

**Table 3.** Activation energies of curing of cardanol polyol/melamine formaldehyde resin mixtures determined by Ozawa method.

*α* %	Activation energy [*E*_a_ (kJ/mol)]				
	70–30	75–25	80–20	85–15	90–10
2	96.52	98.99	108.76	145.61	146.11
10	84.82	87.29	108.93	145.79	145.92
20	85.28	87.75	108.99	146.05	146.02
30	85.55	88.02	109.19	146.25	146.29
40	85.77	88.24	109.27	146.38	146.35
50	85.82	88.29	109.29	146.49	146.44
60	86.00	88.47	109.32	146.99	146.89
70	86.01	88.48	109.73	147.51	147.45
80	86.38	88.85	110.01	147.90	147.79
90	88.32	90.79	112.08	150.62	150.31
100	116.13	117.70	139.29	178.15	179.50

**Table 4.** Kinetic parameter *A* and *A*′ of curing of cardanol polyol/melamine formaldehyde resin mixtures determined by Ozawa method.

*α* %	70–30		75–25		80–20		85–15		90–10	
	*A*′	*A*	*A*′	*A*	*A*′	*A*	*A*′	*A*	*A*′	*A*
2	14.52	−30.16	14.36	−30.22	15.51	−32.07	19.92	−42.03	19.93	−41.22
10	12.75	−25.23	12.59	−25.55	15.43	−31.05	19.87	−40.93	19.87	−40.20
20	12.76	−24.76	12.60	−25.06	15.37	−30.46	19.85	−40.40	19.86	−39.50
30	12.75	−24.26	12.59	−24.70	15.36	−30.04	19.82	−39.79	19.84	−39.00
40	12.74	−23.88	12.58	−24.31	15.33	−29.61	19.80	−39.21	19.81	−38.41
50	12.70	−23.47	12.54	−23.88	15.29	−29.17	19.78	−38.64	19.79	−37.85
60	12.68	−23.02	12.52	−23.49	15.26	−28.67	19.81	−38.17	19.82	−37.31
70	12.64	−22.60	12.48	−23.11	15.27	−28.23	19.85	−37.75	19.86	−36.89
80	12.63	−22.25	12.47	−22.73	15.26	−27.64	19.89	−37.22	19.90	−36.41
90	12.82	−22.34	12.66	−22.73	15.47	−27.72	20.25	−37.22	20.40	−36.32
100	16.21	−31.02	16.35	−30.87	18.90	−36.07	23.50	−46.04	23.40	−45.61

**Figure 8.**
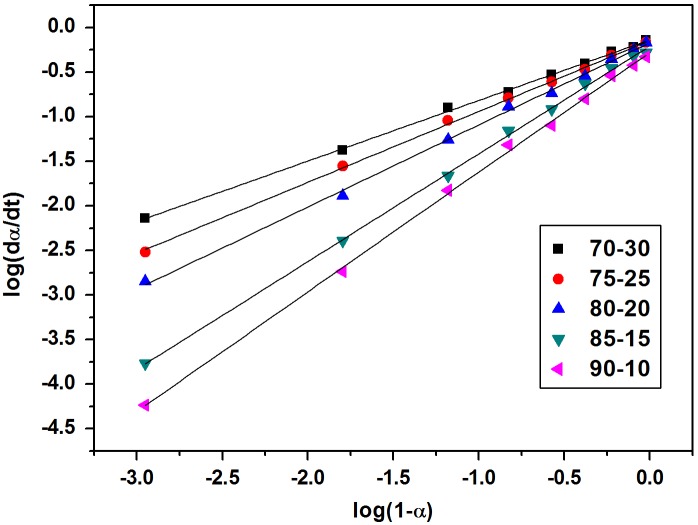
Plots of log (*dα/dt*) vs. log(1 – *α*) for resin mixtures cured at 150 °C.

The reaction order and rate constants for all the curing mixtures were calculated from each slope of log (*dα*/*dt*) vs. log (1 – *α*) and from the intercept respectively as shown in Figure 8. In all cases data fit quite well when assuming the *n*th order reaction kinetic model, since the correlation coefficients of the regression lines are higher than 0.95. Determined values of the rate constants (*k*) and reaction order (*n*) are presented in Table 5. The rate constant increases with increasing the content of melamine resin in mixtures. The reaction order varied from 0.68 to 1.34 for cardanol polyol/melamine formaldehyde resin mixtures. On decreasing the melamine formaldehyde content in the mixtures the values of activation energies (Table 5) was observed to increase from 116.13 to 179.55 kJ/mol. The pre-exponential factors *k*_o_ followed the same trend as that of the activation energies as shown in Table 6. The apparent degree of curing of the resin mixtures is an indicator of the content of reacted functional groups up to a definite time of reaction, at a certain temperature. These data can be used for the estimation of the cross linking degree. The values of apparent degrees of curing after 30 min of reaction for all examined resin mixtures determined by DSC method and by insoluble sol fraction method are given in Table 7. The apparent degree of curing was observed to increase with increasing curing temperature and with ratio of melamine resin in the resin mixtures as shown in Table 7.

**Table 5.** Reaction order (*n*) and rate constants (*k*) of cardanol polyol/melamine formaldehyde resin mixtures cured at 150 °C.

Curing mixtures	*n*	*k* (min^−1^)
70–30	0.68	1.0750
75–25	0.79	0.9500
80–20	0.92	0.6402
85–15	1.21	0.3158
90–10	1.34	0.1466

**Table 6.** Activation energies and pre-exponential factor of resin mixture curing.

Cardanol polyol/melamine formaldehyde resin ratio	*E*_a_ (kJ/mol)	Pre-exponential factor (min^−1^)
70–30	116.13	2.36 × 10^14^
75–25	117.71	3.3 × 10^14^
80–20	139.29	1.02 × 10^17^
85–15	178.15	3.16 × 10^21^
90–10	179.55	2.18 × 10^21^

**Table 7.** Apparent degree of curing of resin mixtures.

Cardanol polyol/melamine formaldehyde resin ratio	Apparent degree of curing (%)							
	By DSC method				By sol-gel method			
	120 °C	130 °C	140 °C	150 °C	120 °C	130 °C	140 °C	150 °C
70–30	78.62	89.06	97.62	99.99	77.41	89.75	97.21	99.87
75–25	74.16	86.78	95.12	99.96	75.34	87.08	95.89	99.89
80–20	67.21	81.78	89.58	99.95	67.94	82.38	89.25	99.87
85–15	58.92	74.71	82.67	99.95	57.98	73.95	81.99	99.83
90–10	51.59	67.71	79.56	99.93	52.12	67.03	80.06	99.84

### 3.4. Coatings film characterization

The curing mixtures of cardanol polyol and melamine formaldehyde resin were evaluated for coating properties at optimized temperature and at optimized time for each ratio obtained from DSC study. The maximum curing conversion was obtained at 150 °C. From the time study, it was observed that on decreasing the melamine formaldehyde content in curing mixtures from 30 to 10 wt%, the time of curing extended from 10 to 30 min. The cured films on mild steel were evaluated for mechanical, optical, chemical and solvent resistance, thermal, and anti-corrosive properties. All the coatings were completely cured, clear with no visible defects such as pin holes, blisters, phase separation, etc. The dry film thickness (DFT) was measured on DFT meter (Positector Standard, USA) and was observed to be in the range of 40–50 μm.

#### 3.4.1. General coating properties

##### 3.4.1.1. Mechanical and optical properties

Gloss of the coatings measured using digital glossometer at 60° was observed to be in the range of 95–98. The adhesion and flexibility of the coatings were investigated by cross-hatch and conical mandrel method respectively. The adhesion and flexibility of coating containing 30 wt% of melamine formaldehyde resin was observed to fail. On decreasing melamine content, the adhesion and flexibility was observed to improve. The higher functionality in polyol along with increased amount of melamine resin (30 wt%) has led to increased brittleness in the coating. The hardness properties (pencil and scratch) of the coating film increased with increasing content of melamine formaldehyde resin in the curing mixture. That can be explained by presence of higher functionality of the melamine resin resulting in formation of a more cross linked network at higher ratio of melamine in the mixture.[32] The impact resistance was evaluated to study the load distribution property of the coating systems. The resistance was measured by falling ball impact method and it was observed to decrease on increasing melamine content. This could be attributed to increased crosslink density and rigid structure of melamine formaldehyde resin. All mechanical properties are reported in Table 8.

**Table 8.** Mechanical properties of cured coatings.

Coatings	Gloss (60°)	Adhesion	Flexibility	Pencil hardness	Scratch hardness (gm)	Impact resistance (inch-pounds)	
						Intrusion	Extrusion
70–30	96 ± 2	0B	17 mm crack	5H	2.6	94.49	15.75
75–25	95 ± 2	3B	6 mm crack	4H	2.4	94.49	31.5
80–20	95 ± 2	5B	0 mm	3H	2.3	94.49	62.99
85–15	96 ± 2	5B	0 mm	3H	2.3	94.49	94.49
90–10	96 ± 2	5B	0 mm	2H	2.1	94.49	94.49

##### 3.4.1.2. Chemical and solvent resistance properties

All the coating systems were evaluated for their acid and alkali resistance by immersion method. The coatings showed excellent resistance to acid (5% HCl) and alkali (5% NaOH) (for 24 h immersion test) without any defects such as blistering or loss of gloss. The hydrolytic stability test was conducted for 24 h also showed very good results in case of all the coatings. This could be attributed to the cross-linked chemical backbone of the films suggesting excellent curing of the coating systems. The good chemical resistance could also be attributed to the heterocyclic ring of melamine formaldehyde resin which imparts excellent chemical stability. Further the solvent resistance of the coatings was also evaluated by solvent rub test. Xylene and methyl ethyl ketone were used as test solvents. In both the cases, the solvent rub test showed no effects on the physical properties of the films even after 200 cycles.

##### 3.4.1.3. Anti-corrosive properties

**Figure 9.**
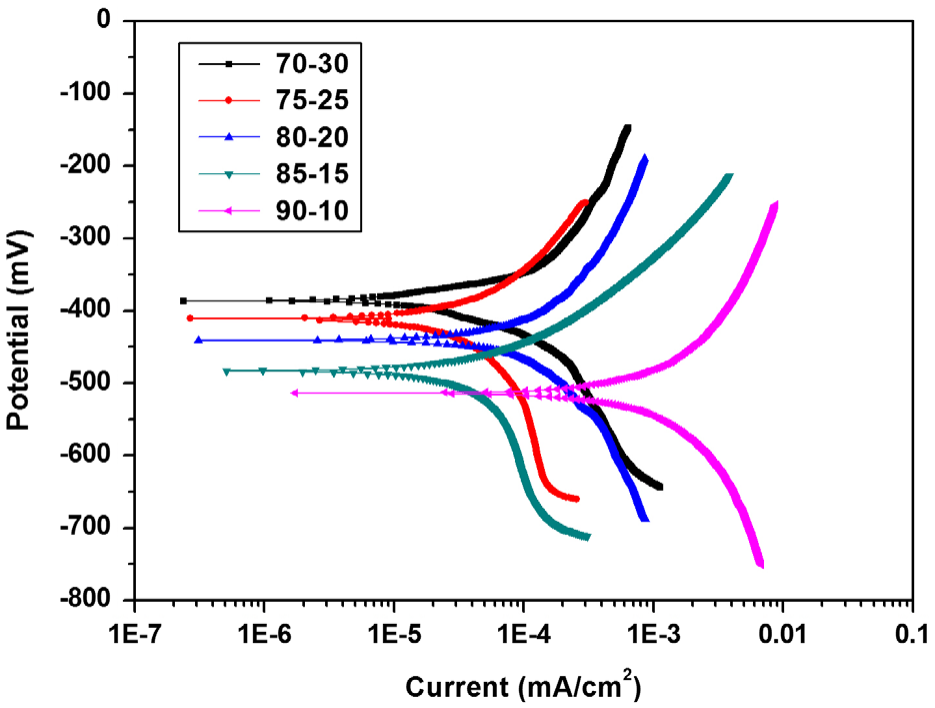
Potentiodynamic polarization curves of cured coatings.

**Figure 10.**
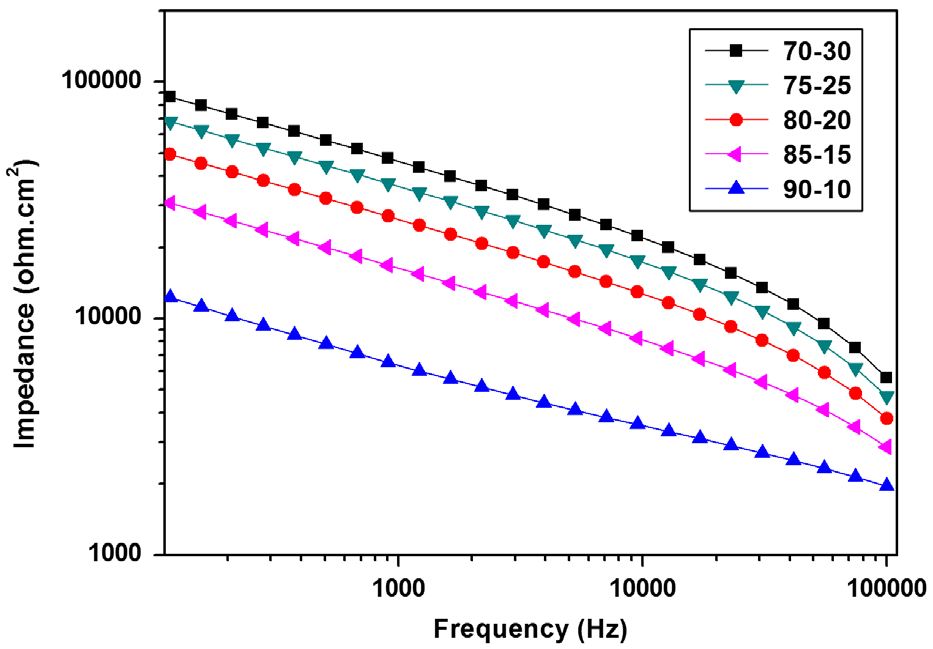
Bode plots of cured coatings.

The potentiodynamic polarization (TAFEL) studies and electrochemical impedance spectroscopy (EIS) was carried out to evaluate the anticorrosive properties of cured coatings. The electrochemical potential was observed to increase with increasing melamine formaldehyde content in the resin mixtures. The corrosion current (*I*_corr_) followed the opposite trend than that of the potential as shown in Figure 9. This indicated that the coating inhibited the anodic process and acted as a barrier to water and chloride ion by impeding its contact with the metal surface.[33] With increasing melamine content in curing mixtures, the crosslinking densities were observed to increase which could improve the anodic inhibition efficiency of the coating in agreement with the lowest corrosion current density and corrosion rate values for 70–30 sample (as shown in Table 9). The impedance values drawn against the frequency in Figure 10 showed the highest value of 9.87 × 10^4^ for 70–30 against other systems, indicating better corrosion resistant coating for the metal substrate. In summary, the results showed that the increased crosslinking density due to increased melamine formaldehyde content was efficient enough to provide excellent corrosion resistance.

**Table 9.** Electrochemical parameters of cardanol polyol cured with MF resin.

Sample name	Impedance (ohms cm^2^)	Corrosion potential (mV)	Corrosion current (mA/cm^2^)	Corrosion rate (mm/year)
70–30	9.87 × 10^4^	−410.67	2.07 × 10^−7^	6.24 × 10^−5^
75–25	7.67 × 10^4^	−426.37	2.4 × 10^−7^	5.71 × 10^−4^
80–20	6.16 × 10^4^	−440.71	3.09 × 10^−7^	7.31 × 10^−3^
85–15	4.49 × 10^4^	−462.53	5.11 × 10^−7^	6.85 × 10^−2^
90–10	1.37 × 10^4^	−513.88	1.71 × 10^−6^	7.66 × 10^−1^

##### 3.4.1.4. Insoluble sol fraction

The insoluble sol fraction of the cured films was measured to study the extent of curing of the coating formulations. The cured films with known initial weight were placed in solvent mixture (xylene: dimethyl formamide: acetone = 50: 25: 25 by v/v) for 24 h. After the test was over, the weight losses of the dried films were measured. The insoluble sol fraction of all the resin mixtures at different isothermal temperatures did not show any significant difference than that of the obtained values from DSC study. The values of curing conversions are reported in Table 7.

##### 3.4.1.5. Thermal properties

**Figure 11.**
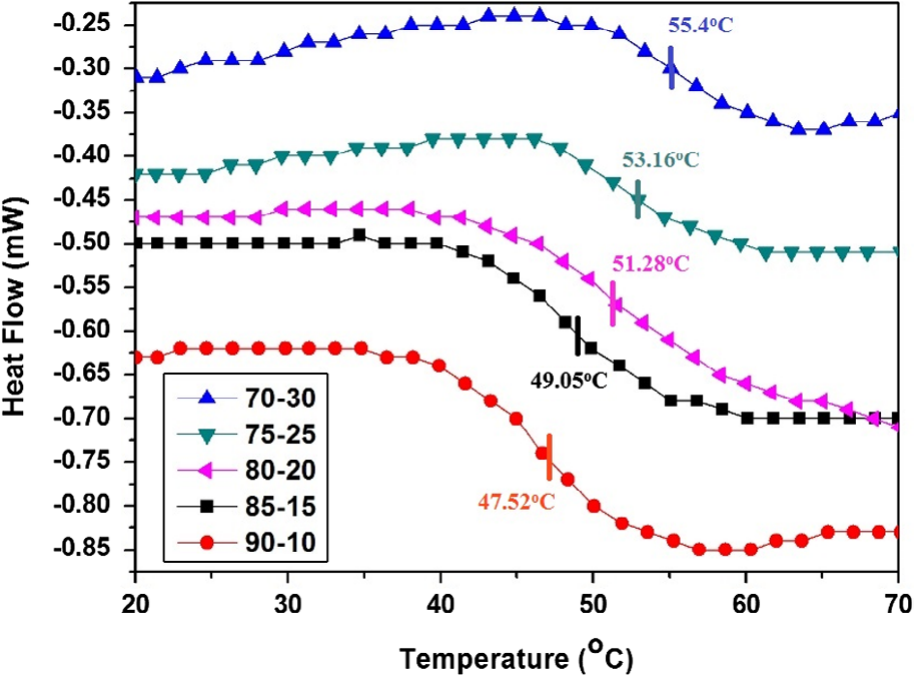
DSC thermographs of all the resin mixtures cured at 150 °C.

The thermal behavior of cured films was investigated with the help of differential scanning calorimeter (DSC) and thermogravimetric (TG) analysis. The polyol with varying melamine content would lead to crosslinked network with variable crosslink densities and directly affect the glass transition temperature. In addition to crosslinking densities, the heterocyclic structure of melamine formaldehyde would largely influence the *T*_g_ of the cured network. The cured coating with higher melamine content resulted in higher *T*_g_ (as shown in DSC thermograms Figure 11).

**Figure 12.**
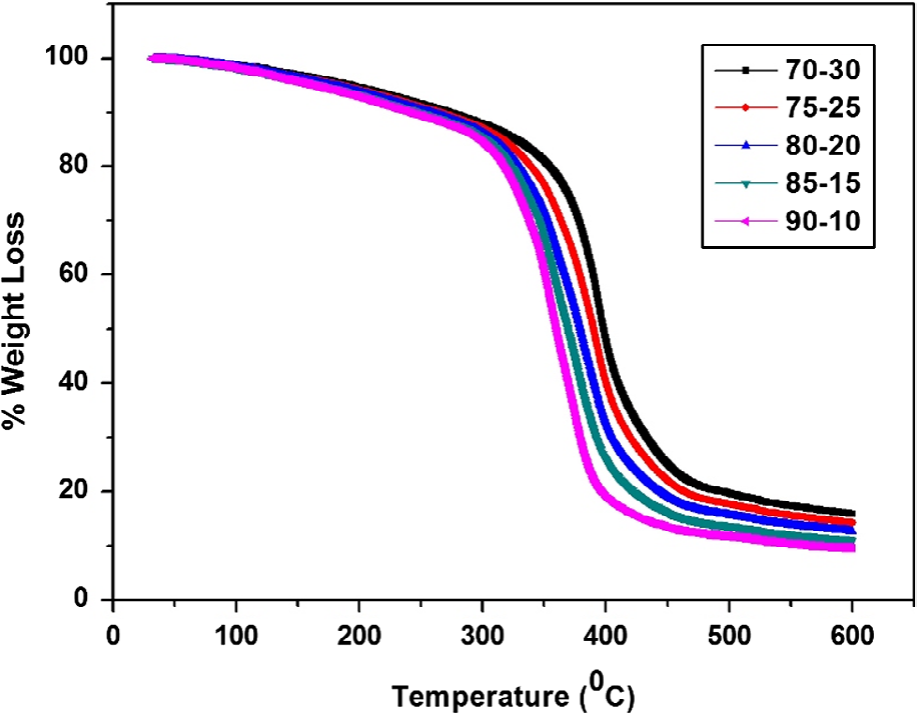
TGA thermographs of all the resin mixtures cured at 150 °C.

The thermal stability of cured coatings mainly depends upon the hard and soft segments in the final structure. The heterocyclic structure of melamine resin used for curing is responsible for the hard segment while long alkyl chains of polyol represented soft segments in the final cured structure. The cured coatings with variable melamine content showed different thermal behavior. It was observed that coatings cured with high melamine content showed high thermal stability which could be attributed to increased number of hard segments [34] as shown in Figure 12. The TGA thermograms of all cured coatings showed 10% degradation (Table 10) starting from 238.62 to 271.56 °C and about 50% decomposition taking place around 360.37 to 398.34 °C. Overall thermal stability of cured coatings showed increasing trend with increasing melamine content in the curing mixture as the rigid segments content increased which supports the literature.[35] The glass transition temperature (*T*_g_) and degradation temperature (*T*_degrad_) values of all coating systems are reported in Table 10.

**Table 10.** Thermal properties of cured coatings.

Sample name	70–30	75–25	80–20	85–15	90–10
*T*_g_ (°C)	55.4	53.2	51.3	49.1	47.5
*T*_10%_	272	264	256	249	239
*T*_50%_	398	390	379	369	360

## 4. Conclusion

Cardanol based polyol was successfully developed, characterized and utilized in preparation of industrial baking enamel. The curing kinetics of developed polyol with melamine formaldehyde resin was investigated by DSC using Ozawa iso-conversional method to calculate the kinetic parameters. The resin mixtures were optimized at different temperature of curing by dynamic DSC scan which was further used in isothermal scan to study the apparent degree of curing vs. time. Depending on melamine content in the curing mixtures, the values of activation energies of curing process were observed from 116.13 to 179.55 kJ/mol. The rate constants were observed to increase with increasing melamine content in the curing mixture. The order of curing reaction varied from 0.68 to 1.34. The value of apparent degree of curing did not significantly vary with methods (DSC and insoluble sol fraction method) applied for its determination. The conversion (apparent degree of curing) was observed to increase with increasing melamine content and with curing temperature. The curing mixtures at optimized temperature and time obtained from DSC scan were further evaluated for coating properties. The content of melamine formaldehyde and subsequently introduced crosslink density were observed to play an important role in deciding the coatings properties. The excellent chemical resistance, good thermal stability and anticorrosive performance suggested that the coatings could be suitably applied on metal substrates for high end applications.

## Funding

This work was supported by the Prime Minister's Fellowship for Doctoral Research [grant number 009-2014].

## Disclosure statement

No potential conflict of interest was reported by the authors.
